# Systematic prioritization of potential therapeutic targets for glomerulonephritis using multi-omics Mendelian randomization

**DOI:** 10.1371/journal.pcbi.1014174

**Published:** 2026-04-16

**Authors:** Guoqiang Li, Fu Jianhan, Jiashu Gu, Yinhuai Wang, Jiachen Liu, Dong Yang, Dianjie Zeng, Pengcheng Zhao

**Affiliations:** 1 Department of Urology, The Affiliated Cancer Hospital of Zhengzhou University & Henan Cancer Hospital, Zhengzhou 450008, China; 2 Department of Urology, Sun yat-sen Memorial Hospital, Sun Yat-sen University, Guangzhou, China; 3 Department of Urology, The Second Xiangya Hospital at Central South University, Changsha, Hunan, China; 4 Xiangya School of Medicine, Central South University, Changsha, Hunan, China; 5 Department of Nephrology, The Second Xiangya Hospital at Central South University, Changsha, Hunan, China; Indonesia International Institute for Life Sciences, INDONESIA

## Abstract

Glomerulonephritis (GN) is an immune-mediated kidney disorder that causes glomerular injury, progressive renal dysfunction, and end-stage kidney disease. Traditional treatments such as corticosteroids and immunosuppressants are limited by variable efficacy and severe adverse effects, highlighting the need for novel therapeutic targets and personalized strategies. We performed a systematic multi-omics Mendelian randomization (MR) analysis applying established proteomic and transcriptomic quantitative trait loci (pQTL/eQTL) resources to genome-wide association studies (GWAS) of four GN subtypes: acute, chronic, IgA nephropathy, and membranous nephropathy. Bayesian colocalization was used to strengthen causal inference, while independent replication and meta-analysis were conducted using the FinnGen cohort. Mouse knockout phenotypes, drug reposition, and computational pharmacology algorithm were applied to evaluate translational potential. Proteomic-wide MR revealed *MTR* as protective in chronic GN and *HCK* as a risk factor for membranous nephropathy, whereas *CD302* and *CDKN1B* showed protective effects. Transcriptomic-wide MR identified candidate genes across GN subtypes: *RECQL*, *BRSK2*, and *MGP* in acute GN; *AFM*, *CFHR5*, and *EPHB2* in chronic GN; *IL6R*, *MBL2*, and *PRSS3* in IgA nephropathy; and *TIMP4*, *HCK*, and *PEAR1* in membranous nephropathy. Bayesian colocalization analysis provided strong support for shared causal variants (PPH4 > 0.8) for *HCK*, *CD302*, *TIMP4*, *PEAR1*, *PARP1*, and *FHIT*. Replication and meta-analysis in the FinnGen cohort provided additional consistency across datasets, while downstream translational annotations highlighted *IL6R*, *MBL2*, *C5*, and *CD55* as potential hub targets within immune and complement-related pathways. This integrative multi-omics study provides novel insights into the genetic architecture and therapeutic landscape of GN, identifying potential therapeutic targets that may inform precision nephrology and drug repurposing. Notably, most targets supported by colocalization, mouse knockout phenotypes, and drug repurposing evidence were predominantly identified in membranous nephropathy, suggesting a particularly tractable genetic and therapeutic architecture for this subtype.

## Author summary

Glomerulonephritis is a group of kidney diseases caused by abnormal immune responses that damage the filtering units of the kidney. Although treatments such as steroids and immunosuppressive drugs are widely used, they do not work equally well for all patients and often cause serious side effects. Identifying safer and more precise treatment targets remains a major challenge. In this study, we used genetic data from large international studies to systematically prioritize genes that may play a role in different types of glomerulonephritis. By combining information on genetic variation, gene activity, and protein levels, we were able to assess whether changes in specific genes are likely to influence disease risk. We also examined whether these genetic signals were consistent across independent datasets and whether the identified genes are already linked to existing drugs or biological pathways. Our results highlight clear differences between disease subtypes. In particular, membranous nephropathy showed the strongest genetic signals linked to immune and complement pathways, suggesting that this form of kidney disease may be especially suitable for targeted therapies. Rather than providing definitive proof, our work offers a prioritized list of candidate targets that can guide future laboratory experiments and clinical research. Overall, our study demonstrates how genetic data can help focus drug discovery efforts and support more precise treatment strategies for immune-related kidney diseases.

## 1. Introduction

Glomerulonephritis (GN) is a group of kidney diseases caused by immune-mediated inflammation, primarily characterized by injury to the glomeruli [1]. The pathological features of GN include proteinuria, hematuria, and progressive renal dysfunction, which can ultimately lead to end-stage renal disease (ESRD) [2]. As a major cause of kidney disease worldwide, GN remains a therapeutic challenge. Traditional treatment strategies, such as corticosteroids, immunosuppressants, and anti-CD20 monoclonal antibodies (e.g., rituximab), can effectively control symptoms in some patients [3–5]. However, these therapies are often limited by severe side effects, such as infections, hypertension, and an increased risk of tumors, as well as by significant inter-individual variability in treatment efficacy [6–8]. Thus, identifying novel drug targets and developing personalized treatment strategies are critical to improving the prognosis of GN patients.

With the rapid advancement of multi-omics technologies, genomics, transcriptomics, and proteomics have provided new perspectives for understanding the mechanisms underlying complex diseases [9,10]. These technologies have not only illuminated the genetic basis of diseases but also provided rich insights for drug target discovery. However, translating this vast amount of data into potentially therapeutic targets remains a significant challenge. Traditional genome-wide association studies (GWAS) have identified genetic variants associated with GN [11], but these studies are often hindered by phenotypic diversity and confounding environmental factors, limiting the ability to draw causal conclusions.

As an effective genetic epidemiological inference method, Mendelian randomization (MR) analysis uses genetic variants as instrumental variables to minimize the impact of environmental and behavioral confounders, thus providing more reliable evidence for drug target identification [12]. Combined with computational pharmacology tools, it not only allows for the assessment of causal relationships between genes and diseases but also integrates large-scale genomic and proteomic data to validate potential therapeutic targets and explore their pharmacological interventions [13].

**Fig 1:**
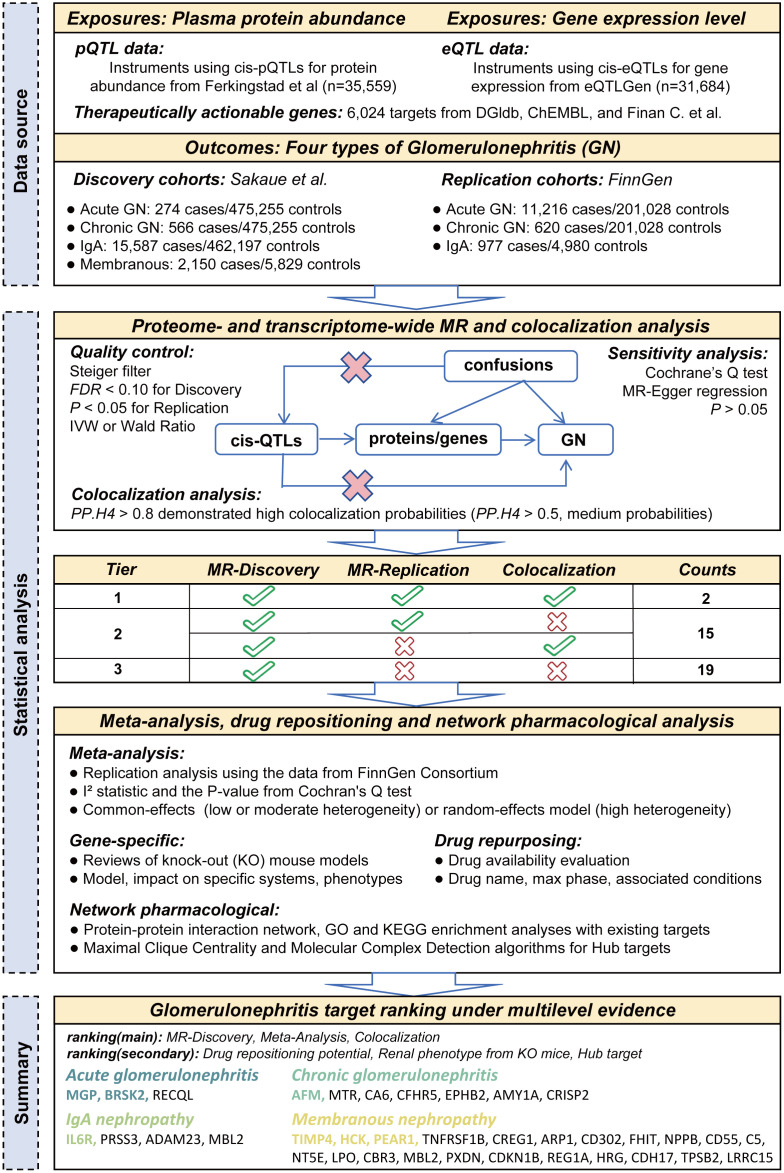
Flowchart of the study design. The figure consists of three sections: data sources, statistical analysis, and summary. In the summary section, the highest-scoring candidate targets for each outcome are highlighted in color.

In this study, we employ such a genetic epidemiological approach based on proteomic and transcriptomic data to systematically prioritize potential drug targets, integrated with quantitative data from multiple databases and computational pharmacology algorithms. We identify genes and proteins with genetic evidence consistent with genetically supported associations in GN, and further assess the plausibility of shared genetic mechanisms using colocalization analysis. Additionally, by applying computational pharmacology tools, we place the prioritized targets in a translational context by integrating druggability annotations and computational pharmacology resources. The primary objective of this study is to offer a novel perspective on the genetic basis of glomerulonephritis by integrating multi-omics data and advanced computational methods, and to provide important references for future drug development and clinical treatment strategies (Fig 1).

## 2. Methods

### 2.1 Data source for glomerulonephritis

We leveraged several large-scale GWAS focusing on glomerulonephritis, derived from multiple datasets published by Sakaue et al [14]. Each dataset included different numbers of individuals with the following diseases and controls of European ancestry: acute glomerulonephritis (274 cases/475,255 controls), chronic glomerulonephritis (566 cases/475,255 controls), IgA nephropathy (15,587 cases/462,197 controls), and membranous nephropathy (2,150 cases/5,829 controls) (S1 Table).

We used summary statistics from the FinnGen Consortium (https://www.finngen.fi/en) for external replication in an independent sample cohort. Each dataset for replication included different numbers of individuals with the following diseases and controls of European ancestry: acute glomerulonephritis (11,216 cases/201,028 controls), chronic glomerulonephritis (620 cases/201,028 controls), and IgA nephropathy (977 cases/4,980 controls) (S1 Table).

### 2.2 Druggable targets and plasma protein/Expression quantitative trait loci (pQTL/eQTL) data

Druggable genes were identified using data from the Drug–Gene Interaction Database (DGIdb, version 4.2.0, https://www.dgidb.org/), specifically the “Categories Data” released in February 2022, which includes all genes classified as druggable and mapped to Entrez genes. In addition, we referred to a list of druggable genes from the review by Finan et al [15].

For plasma protein/expression quantitative trait loci data, we used genetic instrumental variables reported by Ferkingstad et al [10], the largest pQTL analysis to date, covering 4,907 proteins in 35,559 individuals of Icelandic descent. This study identified 18,084 associations between genetic variants and plasma protein levels. pQTLs were extracted from the deCODE study using aria2c (http://aria2.sourceforge.net/). To reduce concerns about horizontal pleiotropy, we restricted instrumental variables to cis-pQTLs (SNPs located within a 1,000 kb window around the target gene) for each protein. For eQTL instruments, genetic variants robustly associated with gene expression within a 1,000 kb region flanking the coding sequence were extracted from eQTL summary statistics provided by the eQTLGene Consortium [9].

### 2.3 Multi-omics Mendelian Randomization (MR) analysis

Mendelian Randomization (MR) relies on three key assumptions: (1) Instrumental variables must be associated with the exposure; (2) Instrumental variables should not be related to potential confounders; (3) Instrumental variables affect the outcome solely through their influence on the exposure [16]. To fulfill these assumptions, we first mapped single-nucleotide polymorphisms (SNPs) to human genome Build 37 (GRCh37) to standardize genomic coordinates. We then rigorously derived instrumental variables from druggable pQTL and eQTL data, applying the following criteria: (1) SNPs associated with each exposure were selected based on genome-wide significance (*P* < 5 × 10 ⁻ ⁸); (2) Cis-acting SNPs (cis-QTLs) within 1,000 kb of transcription start sites for proteins and genes were included, as cis instruments are less likely to violate the horizontal pleiotropy assumption compared to trans instruments [17]; (3) SNPs in the major histocompatibility complex (MHC) region (chr6: 26 Mb to 34 Mb) were excluded due to complex linkage disequilibrium (LD) patterns [17]; (4) LD analysis was performed within a 10,000 kb window to remove SNPs with r² > 0.001; (5) SNP strength was assessed using the F-statistic (mean β²/σ²), where β represents the SNP effect size on the exposure, and σ is the standard error of β. SNPs with F-statistics greater than 10 were retained to minimize weak instrument bias, a crucial aspect in MR studies [18]; (6) SNPs with ambiguous palindromic structures were excluded to avoid uncertainty.

Additionally, each instrumental variable and its proxies were queried in the PhenoScanner GWAS database (http://phenoscanner.medschl.cam.ac.uk) to identify potential associations with confounders such as education, smoking, drinking, blood pressure, and socioeconomic status. SNPs associated with these confounders (*P* < 1 × 10 ⁻ ⁵) were excluded [19]. The remaining SNPs were used for MR analysis. If only one genetic instrumental variable was available for a given protein or gene, the Wald ratio was used. The inverse-variance weighted (IVW) method was applied when two or more instrumental variables were available. Statistical significance was determined using false discovery rate (FDR) correction at a threshold of FDR = 0.05.

### 2.4 Sensitivity analysis

Sensitivity analysis was conducted to assess the robustness of the results. For proteins or genes with more than two instrumental variables, Cochran’s Q was calculated to test for heterogeneity [20]. A significant P-value (P < 0.05) indicates the presence of heterogeneity. If heterogeneity was detected, a random-effects model using the IVW method was applied; otherwise, a fixed-effects IVW method was used. Horizontal pleiotropy was evaluated using MR-Egger regression when three or more instrumental variables were included [21]. Cis-QTLs with a significant MR-Egger regression intercept (*P* < 0.05) were excluded. The MR-PRESSO test was applied to detect and remove outlier SNPs that might bias causal estimates (SNPs with *P* < 0.05 were removed, S2 Table) [22]. To confirm the causal direction, Steiger filtering were performed [23]. SNPs with a Steiger test *P*-value < 0.05 and reversed causal direction (i.e., R²_outcome > R²_exposure) were removed. Both the Two Sample MR and MR-PRESSO packages in R software (version 4.3.1) were utilized to perform MR and sensitivity analysis.

### 2.5 Colocalisation analysis

Colocalisation analysis was performed to investigate whether the associations between specific protein levels or gene expression and glomerulonephritis could be attributed to the same causal genetic variant. This analysis utilized computed Bayes factors and summary association data to test five mutually exclusive hypotheses: (1) no causal SNP for either glomerulonephritis GWAS or QTL (H0); (2) a causal SNP for glomerulonephritis GWAS only (H1); (3) a causal SNP for QTL only (H2); (4) distinct causal SNPs for glomerulonephritis GWAS and QTL (H3); (5) a shared causal SNP for both glomerulonephritis GWAS and QTL (H4). For proteomic or transcriptomic traits where the instrumental variable was located within a cis region (+/- 1 Mb) of the target gene, colocalisation analysis was conducted using coloc package. Based on prior studies [24], we adopted prior probabilities (p_1_ = 1 × 10 ⁻ ⁴, p_2_ = 1 × 10 ⁻ ⁴, and p_12_ = 1 × 10 ⁻ ⁵), and a posterior probability ≥ 0.80 was considered high colocalization probabilities, while ≥ 0.50 is considered to be medium colocalization probabilities.

### 2.6 Replication and meta-analysis based on FinnGen Consortium

To ensure the robustness of significant findings, we conducted replication analysis using the FinnGen Consortium data. MR estimates from different sources were then combined using either common-effects or random-effects meta-analysis methods. The I² statistic was calculated to assess heterogeneity across sources, with values < 25%, 25–75%, and > 75% indicating low, moderate, and high heterogeneity, respectively. The appropriate model was selected based on the I² statistic and the *P*-value from Cochran’s Q test. For low or moderate heterogeneity (*P* > 0.05), common-effects models were used, while random-effects models were applied for high heterogeneity (*P* < 0.05). Sensitivity analysis, using the same criteria as the initial analysis, was performed to test and confirm the robustness of each MR analysis.

### 2.7 Mouse knock-out models for druggable targets

To investigate whether modification of the target affects a phenotype relevant to glomerulonephritis, we queried the Mouse Genome Informatics (MGI, http://www.informatics.jax.org/) database for candidate targets identified in our MR analysis. We focused on systemic abnormalities associated with knock-out (KO) mouse models, documenting phenotypes related to the renal and urinary systems in detail.

### 2.8 Drug repurposing assessment

We utilized the DrugBank database (https://go.drugbank.com/) and the Open Target Platform (https://platform.opentargets.org/) to identify drugs targeting our candidate proteins and assess their repurposing potential. This evaluation included reviewing the drug indications, development stages, and their corresponding targets.

### 2.9 Network pharmacological analysis with existing targets

The ClinicalTrials.gov database (https://clinicaltrials.gov/) was queried to identify drugs in clinical stages or approved for glomerulonephritis, including IgA nephropathy and membranous nephropathy. A protein-protein interaction (PPI) network was constructed using the STRING database (https://cn.string-db.org/) and Cytoscape software to explore potential connections between candidate targets and clinical trial drugs for glomerulonephritis [25]. Gene Ontology (GO) and Kyoto Encyclopedia of Genes and Genomes (KEGG) enrichment analyses were performed to assess the functional and biological relevance of these targets.

Hub targets were identified using the CytoHubba plugin, which evaluates key nodes and fragile motifs in the interactome network through various topological algorithms, including Degree, Edge Percolated Component (EPC), Maximum Neighborhood Component (MNC), and Maximal Clique Centrality (MCC). The top 10 targets, ranked by the MCC algorithm, were selected. To further validate these hub targets, the Molecular Complex Detection (MCODE) algorithm was applied to identify densely connected modules within the PPI network, enhancing the confidence in the final results.

## 3. Results

### 3.1 Proteomic-wide MR analysis results

**Fig 2:**
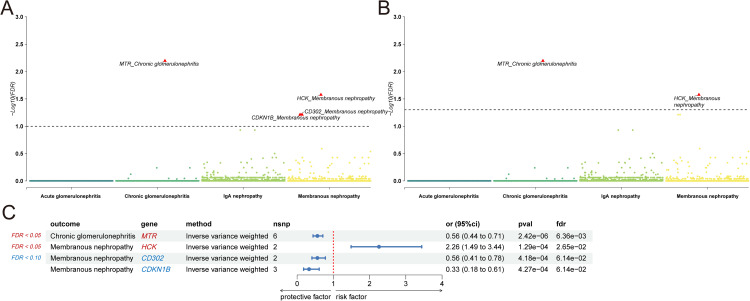
Prioritized proteins for four types of glomerulonephritis identified from proteomics-wide MR analysis. **(A)** Manhattan plot showing the − log10(P.adj value) of association for each protein from the MR plotted on the y-axis against genomic position on the x-axis. The dotted line corresponds to the significance threshold (Benjamini-Hochberg correction, P.adj < 0.10). **(B)** Manhattan plot showing the − log10(P.adj value) of association for each protein from the MR plotted on the y-axis against genomic position on the x-axis. The dotted line corresponds to the significance threshold (Benjamini-Hochberg correction, P.adj < 0.05). **(C)** The forest plot shows detailed results from proteome-wide MR.

We performed a proteome-wide Mendelian randomization (MR) analysis to assess the associations between circulating protein abundance and four types of glomerulonephritis (S3 Table). Statistical significance was evaluated using two false discovery rate (FDR) thresholds (0.05 and 0.10). The overall patterns of association across disease subtypes are summarized in Fig 2A and 2B.

Throughout the results, associations meeting an FDR < 0.05 threshold are considered high-confidence findings, whereas associations identified at FDR < 0.10 are regarded as lower-confidence and exploratory signals. At the proteomic level, significant associations were predominantly observed for chronic glomerulonephritis and membranous nephropathy. In chronic glomerulonephritis, higher genetically predicted plasma levels of methionine synthase (MTR) were associated with a reduced disease risk (FDR < 0.05; OR = 0.56, 95% CI: 0.44–0.71), consistent with a potential protective association (Fig 2C).

In membranous nephropathy, multiple proteins showed evidence of association. Hematopoietic cell kinase (HCK) emerged as a strong risk factor (FDR < 0.05; OR = 2.26, 95% CI: 1.49–3.44), whereas *CD302* and *CDKN1B* displayed protective associations at the FDR < 0.10 threshold (OR = 0.56 and 0.33, respectively). No proteomic associations reached statistical significance for acute glomerulonephritis or IgA nephropathy at either FDR threshold (Fig 2C).

Sensitivity analyses showed no evidence of heterogeneity, horizontal pleiotropy, or influential outliers for the identified associations (S5 and S6 Tables), indicating no major violations of MR assumptions for the identified associations.

### 3.2 Transcriptomic‑wide MR analysis results

**Fig 3:**
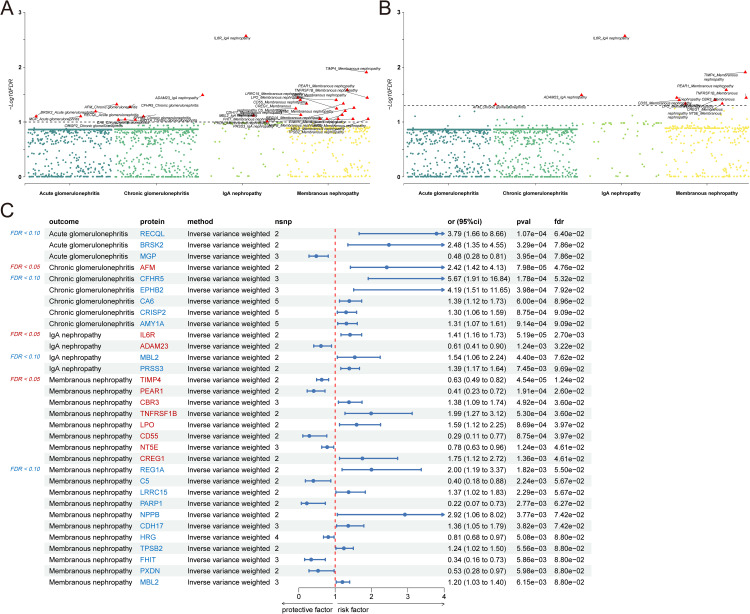
Prioritized genes for four types of glomerulonephritis identified from transcriptome-wide MR analysis. **(A)** Manhattan plot showing the − log10(P.adj value) of association for each gene from the MR plotted on the y-axis against genomic position on the x-axis. The dotted line corresponds to the significance threshold (Benjamini-Hochberg correction, P.adj < 0.10). **(B)** Manhattan plot showing the − log10(P.adj value) of association for each gene from the MR plotted on the y-axis against genomic position on the x-axis. The dotted line corresponds to the significance threshold (Benjamini-Hochberg correction, P.adj < 0.05). **(C)** The forest plot shows detailed results from transcriptome-wide MR.

Transcriptome-wide MR analysis was conducted to evaluate the genetically proxied associations between gene expression and four glomerulonephritis subtypes (S4 Table). Two FDR thresholds (0.05 and 0.10) were applied to identify significant associations, and the overall results are visualized in Fig 3A and 3B.

For acute glomerulonephritis, no transcripts met the FDR < 0.05 threshold. At FDR < 0.10, three genes showed evidence of association, including *RECQL* and *BRSK2* as risk factors and MGP as a protective factor, suggesting that transcriptional regulation may contribute to acute disease susceptibility (Fig 3C).

In chronic glomerulonephritis, *AFM* was the only transcript reaching the FDR < 0.05 threshold, indicating an increased risk. Several additional genes, including *CFHR5* and *EPHB2*, were associated at the FDR < 0.10 level, pointing toward complement-related and receptor-mediated pathways (Fig 3C).

For IgA nephropathy, *IL6R* and *ADAM23* showed significant associations at FDR < 0.05, while *MBL2* and *PRSS3* met the FDR < 0.10 criterion, highlighting the involvement of cytokine signaling and the lectin complement pathway (Fig 3C).

Membranous nephropathy exhibited the largest number of transcriptomic associations. Several genes reached the FDR < 0.05 threshold, including *TIMP4*, *PEAR1*, *TNFRSF1B*, *LPO*, *CD55*, *NT5E*, and *CREG1*, with additional candidates identified at FDR < 0.10 (Fig 3C). These results indicate a dense network of transcriptionally regulated risk and protective factors in membranous nephropathy.

Across all transcriptomic analyses, sensitivity tests revealed no substantial heterogeneity, horizontal pleiotropy, or influential outliers (S7 and S8 Tables), supporting the consistency of the MR estimates across sensitivity analyses.

### 3.3 Colocalisation analysis

The analysis was performed to evaluate five mutually exclusive hypotheses regarding the genetic architecture underlying the observed associations between QTLs and glomerulonephritis, ranging from no causal variants for either trait (H0) to a shared causal variant affecting both the molecular trait and disease risk (H4). In the Results, we primarily focused on the posterior probability of hypothesis 4 (PPH4), which represents evidence that the association signals for protein levels or gene expression and glomerulonephritis are driven by the same causal genetic variant. A PPH4 greater than 0.8 was considered strong evidence of colocalization, while values between 0.5 and 0.8 were interpreted as moderate evidence.

**Fig 4:**
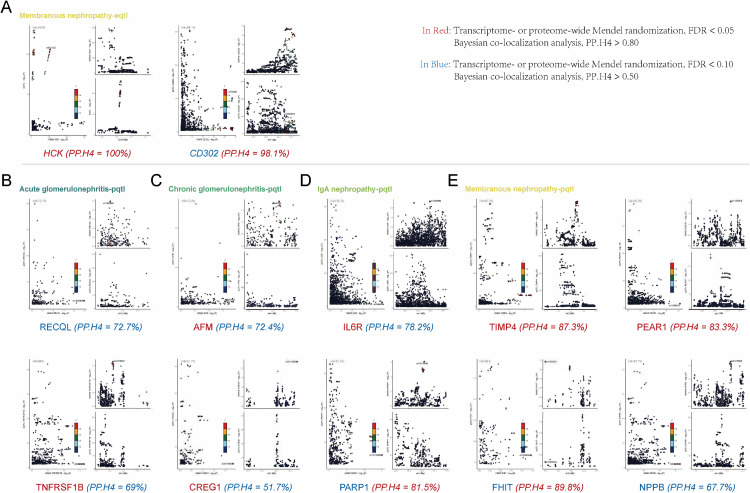
Colocalisation evidence on the associations between identified targets and the glomerulonephritis. **(A)** Colocalization result of membranous nephropathy (eQTL). **(B)** Colocalization result of acute glomerulonephritis (pQTL). **(C)** Colocalization result of chronic glomerulonephritis (pQTL). **(D)** Colocalization result of IgA nephropathy (pQTL). **(E)** Colocalization result of membranous nephropathy (pQTL).

For acute glomerulonephritis, the analysis revealed moderate colocalization evidence for *RECQL* (PPH4 = 72.7%), suggesting that the observed association may be driven by a shared genetic signal. In chronic glomerulonephritis, *AFM* showed moderate colocalization (PPH4 = 72.4%), indicating a potential genetic overlap with chronic kidney injury. For IgA nephropathy, *IL6R* exhibited moderate colocalization with a PPH4 of 78.2%. Finally, in membranous nephropathy, the results were more striking. *HCK* (PPH4 = 100%) and *CD302* (PPH4 = 98.1%) demonstrated strong colocalization evidence, indicating a clear genetic overlap with membranous nephropathy. Additionally, several other genes, including *TIMP4* (PPH4 = 87.3%), *PEAR1* (PPH4 = 83.3%), *PARP1* (PPH4 = 81.5%), and *FHIT* (PPH4 = 89.8%), also showed strong colocalization evidence, suggesting that these genes share genetic signals with disease risk. Other genes such as *TNFRSF1B* (PPH4 = 69%), *CREG1* (PPH4 = 51.7%), and *NPPB* (PPH4 = 67.7%) provided moderate colocalization evidence, warranting further investigation into their role in membranous nephropathy (Fig 4).

### 3.4 Replication and meta-analysis based on FinnGen Consortium

**Fig 5:**
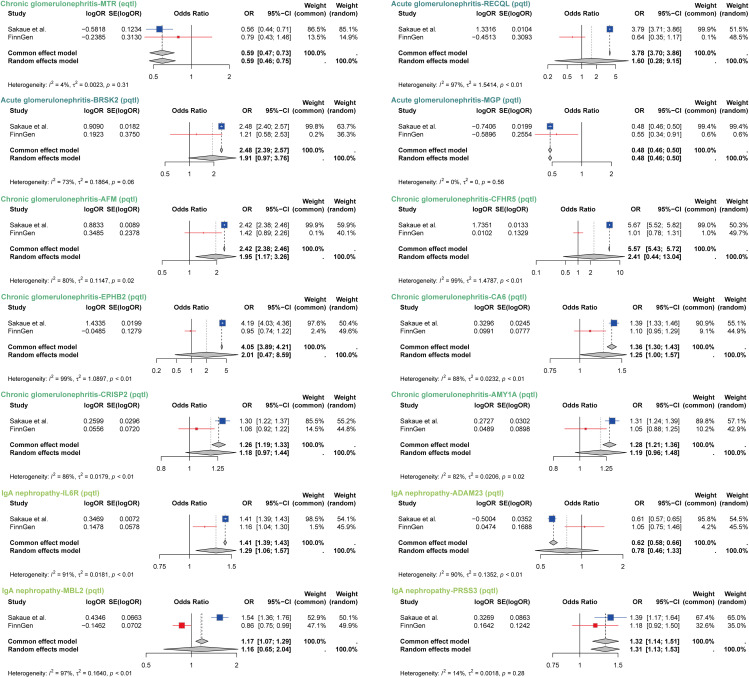
Replication and meta-analysis based on FinnGen consortium. MR estimates from different sources were combined using either common-effects or random-effects meta-analysis methods. For low or moderate heterogeneity (Cochran’s Q test-P > 0.05), common-effects models were used, while random-effects models were applied for high heterogeneity (Cochran’s Q test-P < 0.05).

To validate our findings, we conducted a replication and meta-analysis using data from the FinnGen Consortium. The appropriate statistical models were selected based on the I² statistic and the P-value of Cochran’s Q test, to assess consistency of the MR estimates across independent dataset (P-value > 0.05, common-effects model; P-value < 0.05, random-effects model). For acute glomerulonephritis, the analysis revealed significant findings with *BRSK2*, showing an odds ratio (OR) of 2.48 [2.39–2.57] under the common-effects model, and *MGP*, with an OR of 0.48 [0.46–0.50], also under the common-effects model. In chronic glomerulonephritis, *MTR* was associated with an OR of 0.59 [0.47–0.73] under the common-effects model, suggesting a protective role. Conversely, *AFM* (OR: 1.95 [1.17–3.26]) and *CA6* (OR: 1.25 [1.00–1.57]) were associated with higher risks, with *AFM* exhibiting a random-effects model and *CA6* showing evidence under the same model. For IgA nephropathy, *IL6R* was associated with an OR of 1.29 [1.06–1.57] under the random-effects model, indicating a modest increase in risk, while *PRSS3* showed a positive association with an OR of 1.32 [1.14–1.51] under the common-effects model (Fig 5). These results provide additional consistency with the primary analysis across datasets.

### 3.5 Mouse knock-out models for druggable targets

Mouse knock-out models involving the genes *MGP*, *CDKN1B*, *TIMP4*, *LPO*, *CD55*, *NT5E*, and *PARP1* exhibited a range of phenotypes, including abnormalities in kidney vasculature morphology, increased kidney weight, and altered renal glomerular structure. Additionally, these models showed abnormal kidney morphology, smaller kidney size, glomerulonephritis, and albuminuria. Further findings included abnormal renal vascular resistance, decreased renal tubule apoptosis, reduced susceptibility to kidney reperfusion injury, and an increased renal glomerular filtration rate (Table 1). These renal phenotypes in knockout models are consistent with potential involvement of these genes in renal biology and provide supporting biological context for the genetically prioritized targets.

**Table 1:** Reviews of knock-out mouse models for candidate targets from the MGI database.

Disease	Gene	Model	Impact on specific systems	Phenotypes	MGI ID
Acute glomerulonephritis	*RECQL*	Targeted (Null/knockout)	cellular	abnormal chromosome morphology elevated level of mitotic sister chromatid exchange decreased fibroblast proliferation spontaneous chromosome breakage	3701259
	*BRSK2*	Targeted (Null/knockout)	N/A	N/A	3575113
	*MGP*	Targeted (Null/knockout)	mortality/aging cardiovascular system renal/urinary system	premature death abnormal aorta tunica media morphology abnormal kidney vasculature morphology increased kidney weight abnormal renal glomerulus morphology small kidney	1934912
Chronic glomerulonephritis	*MTR*	Targeted (Null/knockout)	homeostasis/metabolism mortality/aging	embryonic lethality, complete penetrance	2180496
	*AFM*	Targeted (Conditional ready, Null/knockout, Reporter)	behavior/neurological hematopoietic system limbs/digits/tail	abnormal coping response increased hemoglobin content oligodactyly	4455621
	*EPHB2*	Endonuclease-mediated (Null/knockout)	hematopoietic system behavior/neurological	enlarged spleen small spleen increased exploration in new environment	6276938
		Targeted (Null/knockout, Reporter)	digestive/alimentary system renal/urinary system reproductive system	enlarged spleen small spleen increased exploration in new environment	2149670
	*CA6*	Endonuclease-mediated (Null/knockout)	reproductive system immune system craniofacial limbs/digits/tail	abnormal testis morphology abnormal thymus morphology abnormal tooth color abnormal digit morphology	7425557
		Targeted (Null/knockout)	digestive/alimentary system immune system	abnormal Peyer’s patch follicle morphology	5013650
	*CRISP2*	Targeted (Conditional ready, Hypomorph, Null/knockout, Reporter)	reproductive system	abnormal sperm motility reduced male fertility impaired acrosome reaction	4888856
	*AMY1A*	Targeted (Null/knockout)	N/A	N/A	5468001
IgA nephropathy	*IL6R*	Targeted (Null/knockout)	immune system liver/biliary system	decreased circulating tumor necrosis factor level decreased circulating interleukin-1 beta level liver inflammation	5550551
		Targeted (Null/knockout, Reporter)	hematopoietic system	abnormal CD4-positive, alpha-beta T cell physiology	4454682
	*ADAM23*	Targeted (Null/knockout)	mortality/aging behavior/neurological	postnatal lethality, complete penetrance tremors	5613205
		Targeted (Null/knockout, Reporter)	mortality/aging	preweaning lethality, incomplete penetrance	5636978
	*MBL2*	Endonuclease-mediated (Null/knockout)	N/A	N/A	6140211
	*PRSS3*	Endonuclease-mediated (Null/knockout)	N/A	N/A	7291850
Membranous nephropathy	*HCK*	Targeted (Null/knockout)	immune system	impaired macrophage phagocytosis	2384049
	*CD302*	Targeted (Null/knockout)	hematopoietic system immune system	decreased dendritic cell number abnormal CD8-positive, alpha-beta T cell physiology abnormal dendritic cell physiology	6724312
	*CDKN1B*	Targeted (Null/knockout)	renal/urinary system reproductive system adipose tissue	increased kidney weight abnormal prostate gland dorsolateral lobe morphology increased ovary tumor incidence increased brown adipose tissue amount increased white fat cell number	1888808
		Targeted (Null/knockout)	endocrine/exocrine glands immune system	adrenal medulla hyperplasia enlarged adrenal glands enlarged thymus enlarged spleen increased T cell number	1888790
	*TIMP4*	Endonuclease-mediated (Null/knockout)	renal/urinary system hematopoietic system behavior/neurological	small kidney small spleen increased freezing behavior	6276973
		Targeted (Null/knockout)	mortality/aging cardiovascular system renal/urinary system	increased susceptibility to induced morbidity/mortality increased heart left ventricle weight abnormal myocardial fiber physiology decreased coronary flow rate	3663251
	*PEAR1*	Targeted (Conditional ready, Null/knockout, Reporter)	homeostasis/metabolism hematopoietic system	decreased platelet aggregation decreased circulating serum albumin level decreased circulating total protein level	4363617
	*CBR3*	Targeted (Null/knockout, Reporter)	N/A	N/A	4399355
	*TNFRSF1B*	Targeted (Null/knockout)	immune system nervous system homeostasis/metabolism	microgliosis microgliosis decreased susceptibility to dopaminergic neuron neurotoxicity	1860087
		Targeted (Null/knockout)	immune system cellular	decreased T cell proliferation decreased interferon-gamma secretion decreased interleukin-2 secretion	1857262
	*LPO*	Endonuclease-mediated (Null/knockout)	renal/urinary system neoplasm immune system mortality/aging	glomerulonephritis increased tumor incidence increased inflammatory response premature death	7331667
		Targeted (Null/knockout, Reporter)	vision/eye	abnormal lens morphology	5548663
	*CD55*	Targeted (Null/knockout)	behavior/neurological immune system	decreased grip strength abnormal complement pathway increased susceptibility to experimental autoimmune myasthenia gravis myositis	2387405
		Targeted (Null/knockout)	renal/urinary system immune system	albuminuria abnormal podocyte morphology increased susceptibility to experimental autoimmune myasthenia gravis	2383980
	*NT5E*	Endonuclease-mediated (Null/knockout)	renal/urinary system endocrine/exocrine glands hematopoietic system vision/eye	abnormal kidney morphology urinary bladder obstruction abnormal testis morphology abnormal spleen morphology abnormal eye morphology	6449045
		Targeted (Null/knockout)	renal/urinary system homeostasis/metabolism	abnormal renal vascular resistance increased circulating alkaline phosphatase level abnormal response/metabolism to endogenous compounds	3054787
	*CREG1*	Endonuclease-mediated (Null/knockout)	N/A	N/A	7792848
	*REG1A*	Targeted (Null/knockout)	endocrine/exocrine glands	abnormal pancreas physiology	2446480
	*LRRC15*	Targeted (Null/knockout, Reporter)	behavior/neurological cardiovascular system homeostasis/metabolism	decreased thigmotaxis increased heart weight improved glucose tolerance	5493277
	*PARP1*	Targeted (Null/knockout)	renal/urinary system nervous system homeostasis/metabolism	decreased renal tubule apoptosis decreased susceptibility to kidney reperfusion injury increased renal glomerular filtration rate decreased neuron apoptosis abnormal microglial cell physiology decreased circulating creatinine level abnormal response/metabolism to endogenous compounds	1857862
		Endonuclease-mediated (Null/knockout)	vision/eye immune system reproductive system	abnormal eye morphology abnormal spleen morphology abnormal seminal vesicle morphology	7425896
	*NPPB*	Targeted (Null/knockout)	cardiovascular system cellular	abnormal myocardial fiber morphology abnormal heart ventricle morphology cardiac fibrosis disorganized myocardial fiber myofibrils	2386215
	*CDH17*	Targeted (Null/knockout)	immune system hematopoietic system	increased pro-B cell number decreased B cell number decreased spleen germinal center size abnormal spleen marginal zone morphology	3573870
	*HRG*	Targeted (Null/knockout)	hematopoietic system homeostasis/metabolism immune system	increased monocyte cell number increased circulating fibrinogen level abnormal thrombolysis decreased bleeding time	3789962
	*TPSB2*	Targeted (Null/knockout)	immune system	abnormal mast cell differentiation impaired neutrophil chemotaxis impaired eosinophil recruitment decreased susceptibility to induced arthritis increased susceptibility to parasitic infection	3783331
	*FHIT*	Targeted (Null/knockout, Reporter)	mortality/aging immune system neoplasm	premature death decreased granulocyte number increased susceptibility to infection increased tumor incidence	2176611
		Targeted (Null/knockout)	digestive/alimentary system neoplasm	abnormal small intestine morphology increased hepatocellular carcinoma incidence increased lymphoma incidence increased papilloma incidence	3057316
	*PXDN*	Endonuclease-mediated (Null/knockout)	behavior/neurological mortality/aging vision/eye	decreased thigmotaxis preweaning lethality, incomplete penetrance cornea opacity	5829376

### 3.6 Drug repurposing assessment

Upon searching the DrugBank database and Open Target Platform, we identified drugs targeting 23 unique candidate genes or proteins prioritized by the MR analyses (Table 2). In total, these targets were linked to 76 unique drugs that are either approved or under investigation, reflecting that several candidate genes are targeted by multiple pharmacological compounds. Notably, Ravulizumab (targeting *C5*) has been approved for the treatment of atypical hemolytic uremic syndrome (aHUS). Additionally, several other drugs are approved for the prevention or treatment of various conditions, including tumors, nutritional deficiencies, psychiatric disorders, bronchial asthma, and more (Table 2).

**Table 2:** Potential drugs in the DrugBank and Open Target Platform.

Disease	Gene	Drug Name	Drugbank ID	Max Phase	Associated Conditions
Acute glomerulonephritis	*RECQL*	Adenosine 5’-[gamma-thio]triphosphate	DB02930	Preclinical	N/A
	*MGP*	Calcium	DB01373	Approved	Calcium deficiency Deficiency, vitamin d
Chronic glomerulonephritis	*MTR*	Cyanocobalamin	DB00115	Approved	Anemia, pernicious Combined vitamin b1 and b12 deficiency
		Hydroxocobalamin	DB00200	Approved	B vitamin deficiency
		Levomefolic acid	DB11256	Approved	folate deficiency Nutritional supplementation
		Mecobalamin	DB03614	Approved	Vitamin b12 deficiency
		Methionine	DB00134	Approved	Hepatic encephalopathy (he)
		Thimerosal	DB11590	Approved	Skin disinfection
	*AFM*	Copper	DB09130	Approved	supplementation of total parenteral nutrition contraception with intrauterine devices
	*EPHB2*	Edaxeterkib	DB21490	Preclinical	N/A
		Fostamatinib	DB12010	Approved	Chronic immune thrombocytopenia
		Oleandrin	DB12843	Preclinical	N/A
		Phosphoaminophosphonic Acid-Adenylate Ester	DB04395	Preclinical	N/A
		Vandetanib	DB05294	Approved	Metastatic medullary thyroid cancer Locally advanced medullary thyroid cancer
		Ulixertinib	DB13930	Preclinical	N/A
	*CA6*	Cianidanol	DB14086	Approved	Weight Loss
		Curcumin	DB11672	Approved	N/A
		Mafenide	DB06795	Approved	adjunctive topical antimicrobial agent to control bacterial infection
		Sulthiame	DB08329	Approved	Epilepsy, rolandic
	*AMY1A*	4,6-Dideoxyglucose	DB01841	Preclinical	N/A
		6-Amino-4-Hydroxymethyl-Cyclohex-4-Ene-1,2,3-Triol	DB02120	Preclinical	N/A
		Beta-D-Glucose	DB02379	Preclinical	N/A
IgA nephropathy	*IL6R*	Sarilumab	DB11767	Approved	Active polyarticular juvenile idiopathic arthritis (pjia) Polymyalgia rheumatica (pmr) Moderate, active rheumatoid arthritis
		Satralizumab	DB15762	Approved	Neuromyelitis optica spectrum disorder
		Tocilizumab	DB06273	Approved	Coronavirus disease 2019 (covid-19) Cytokine release syndrome caused by car-t cell therapy Giant cell arteritis (gca)
		Vobarilizumab	DB14891	Preclinical	N/A
		Levilimab	DB16387	Preclinical	N/A
	*ADAM23*	Pidolic acid	DB03088	Approved	Acne nos Dry skin
		Ilomastat	DB02255	Preclinical	N/A
		Furoyl-Leucine	DB02215	Preclinical	N/A
		Batimastat	DB03880	Preclinical	N/A
	*MBL2*	alpha-L-methyl-fucose	DB03879	Preclinical	N/A
		Alpha-Methyl-N-Acetyl-D-Glucosamine	DB04426	Preclinical	N/A
		Methyl alpha-D-mannoside	DB01979	Preclinical	N/A
		Methyl beta-L-fucopyranoside	DB03194	Preclinical	N/A
	*PRSS3*	Guanidine-3-propanol	DB03637	Preclinical	N/A
		Benzamidine	DB03127	Preclinical	Gingival disorders nec Gingivitis Infection Inflammation of mouth
		4-Hydroxybutan-1-Aminium	DB02541	Preclinical	N/A
		1,3,2-Dioxaborolan-2-Ol	DB04369	Preclinical	N/A
Membranous nephropathy	*HCK*	Bosutinib	DB06616	Approved	Accelerated phase chronic myelogenous leukemia (cml) Chronic phase chronic myeloid leukemia Blast phase chronic myelocytic leukemia
		Fostamatinib	DB12010	Approved	Chronic immune thrombocytopenia
		Dasatinib	DB01254	Approved	Accelerated phase chronic myologenic leukemia Acute lymphoblastic leukemia Chronic phase chronic myeloid leukemia Myeloid leukemia, chronic, chronic phase
		PP-121	DB08052	Preclinical	N/A
		Quercetin	DB04216	Preclinical	N/A
	*CBR3*	Daunorubicin	DB00694	Approved	Acute lymphoblastic leukaemias (all) Acute myeloid leukemia with myelodysplasia-related changes Ewing’s tumor Lymphoma, diffuse
		Deutetrabenazine	DB12161	Approved	Chorea Tardive dyskinesia (td)
		Doxorubicin	DB00997	Approved	Aids-related kaposi’s sarcoma Acute lymphoblastic leukemia (all) Acute myeloid leukemia Advanced endometrial cancer
		Oxcarbazepine	DB00776	Approved	Partial onset seizures
	*TNFRSF1B*	Tasonermin	DB11626	Approved	Irresectable soft tissue sarcoma of the limb
	*CD55*	Chloramphenicol	DB00446	Approved	Acne Bacterial conjunctivitis Bacterial infections Bacterial dacryocystitis
	*NT5E*	Caffeine	DB00201	Approved	Apnea of prematurity Bronchopulmonary dysplasia (bpd) Common cold
		Cytarabine	DB00987	Approved	Acute lymphocytic leukemia Acute myeloid leukemia Acute promyelocytic leukemia Lymphomatous meningitis
		Dyphylline	DB00651	Approved	Bronchial asthma Bronchospasm
		Enprofylline	DB00824	Approved	asthma peripheral vascular diseases cerebrovascular insufficiency, sickle cell disease diabetic neuropathy.
		Pentoxifylline	DB00806	Approved	Alcoholic liver disease Intermittent claudication Venous leg ulcer
	*REG1A*	Dexfosfoserine	DB04522	Preclinical	Fatigue
		N-acetyl-alpha-neuraminic acid	DB03721	Preclinical	N/A
	*C5*	Avacincaptad pegol	DB15165	Approved	Dry macular degeneration
		Eculizumab	DB01257	Approved	Generalized myasthenia gravis Neuromyelitis optica spectrum disorder Paroxysmal nocturnal haemoglobinuria (pnh) Thrombotic microangiopathies
		Ravulizumab	DB11580	Approved	Atypical hemolytic uremic syndrome (ahus) Myasthenia gravis, generalized Neuromyelitis optica spectrum disorders Paroxysmal nocturnal haemoglobinuria (pnh)
		Pozelimab	DB15218	Approved	Chaple disease
		Zilucoplan	DB15636	Approved	Myasthenia gravis Generalized myasthenia gravis
	*PARP1*	Azacitidine	DB00928	Approved	Acute myeloid leukemia Chronic myelomonocytic leukemia Refractory anemia
		Cladribine	DB00242	Approved	Chronic lymphocytic leukemia Cutaneous t-cell lymphoma Hairy cell leukemia Non-hodgkin’s lymphoma Relapsing multiple sclerosis (rms)
		Niraparib	DB11793	Approved	Advanced epithelial ovarian cancer Advanced primary peritoneal carcinoma Metastatic castration-resistant prostate cancer (mcrpc)
		Zinc acetate	DB14487	Approved	Itch Skin irritation Oozing and weeping
		3-Methoxybenzamide	DB03073	Preclinical	N/A
	*NPPB*	Carvedilol	DB01136	Approved	Atrial fibrillation Chronic stable angina pectoris Hypertension Mild heart failure
	*HRG*	Zinc chloride	DB14533	Approved	a common zinc supplement in parenteral nutrition
		Zinc sulfate, unspecified form	DB14548	Approved	a common zinc supplement in parenteral nutrition
	*TPSB2*	[4-(3-AMINOMETHYL-PHENYL) -PIPERIDIN-1-YL]-(5-PHENETHYL- PYRIDIN-3-YL) -METHANONE	DB04764	Preclinical	N/A
		4-PIPERIDIN-4-YLBUTANAL	DB04654	Preclinical	N/A
		Amido Phenyl Pyruvic Acid	DB02018	Preclinical	N/A
		JNJ-27390467	DB06962	Preclinical	N/A
	*FHIT*	Adenosine monotungstate	DB02373	Preclinical	N/A
		Ado-P-Ch2-P-Ps-Ado	DB04389	Preclinical	N/A
		Fructose	DB04173	Approved	Nausea Upset stomach

### 3.7 Network pharmacological analysis with existing targets

**Fig 6:**
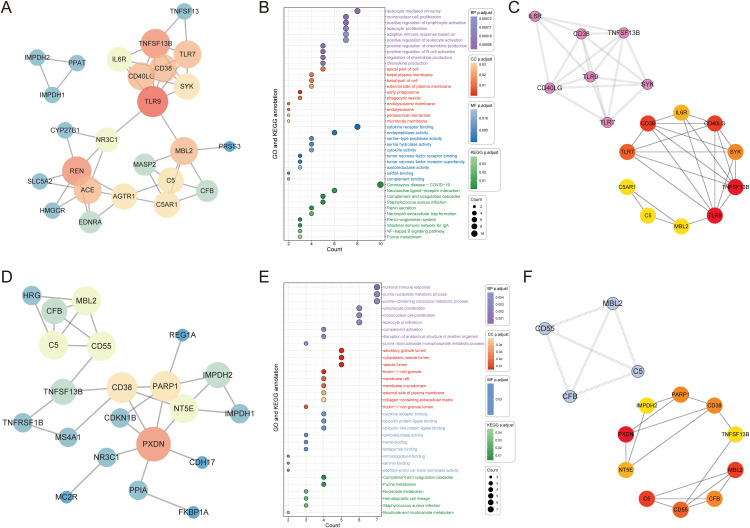
Network pharmacological analysis and identification of hub targets. **(A)** Interaction network diagram between existing drug targets in IgA nephropathy and newly identified targets in this study. **(B)** GO and KEGG pathway enrichment analysis. **(C)** Maximal Clique Centrality (MCC) and Molecular Complex Detection (MCODE) algorithms were used to identify hub targets in IgA nephropathy. **(D)** Interaction network diagram between existing drug targets in membranous nephropathy and newly identified targets in this study. **(E)** GO and KEGG pathway enrichment analysis. **(F)** Maximal Clique Centrality (MCC) and Molecular Complex Detection (MCODE) algorithms were used to identify hub targets in membranous nephropathy.

Through the ClinicalTrials.gov database, we identified 34 potential drugs undergoing clinical trials for IgA nephropathy, corresponding to 24 unique targets, and 12 potential drugs undergoing clinical trials for membranous nephropathy, corresponding to 10 unique targets (S9 and S10 Tables). Using the STRING database and Cytoscape software, we constructed protein interaction networks encompassing all candidate targets and corresponding experimental drug targets for nephropathies (Fig 6A and 6D). Newly identified targets such as *MBL2* and *PXDN* exhibited strong interactions with existing drug targets. Based on these targets, we performed GO and KEGG enrichment analyses, revealing the most highly enriched biological pathways. Candidate targets for IgA nephropathy were predominantly enriched in pathways including leukocyte-mediated immunity, mononuclear cell proliferation, and positive regulation of lymphocyte activation (Fig 6B). Candidates for membranous nephropathy showed enrichment in humoral immune response, purine nucleotide metabolic processes, and lymphocyte proliferation (Fig 6E).

Different algorithms were employed to identify hub targets for glomerulonephritis. In IgA nephropathy, *IL6R* was identified by both MCC and MCODE algorithms, while *MBL2* was identified by the MCC algorithm (Fig 6C). In membranous nephropathy, *MBL2*, *C5*, and *CD55* were identified by both MCC and MCODE algorithms, while *PXDN*, *PARP1*, and *NT5E* were identified by the MCC algorithm (Fig 6F). The observed network connectivity provides biological context and helps place the prioritized targets within pathways relevant to existing therapeutic strategies.

### 3.8 Genetic evidence-based prioritization of candidate targets

**Fig 7:**
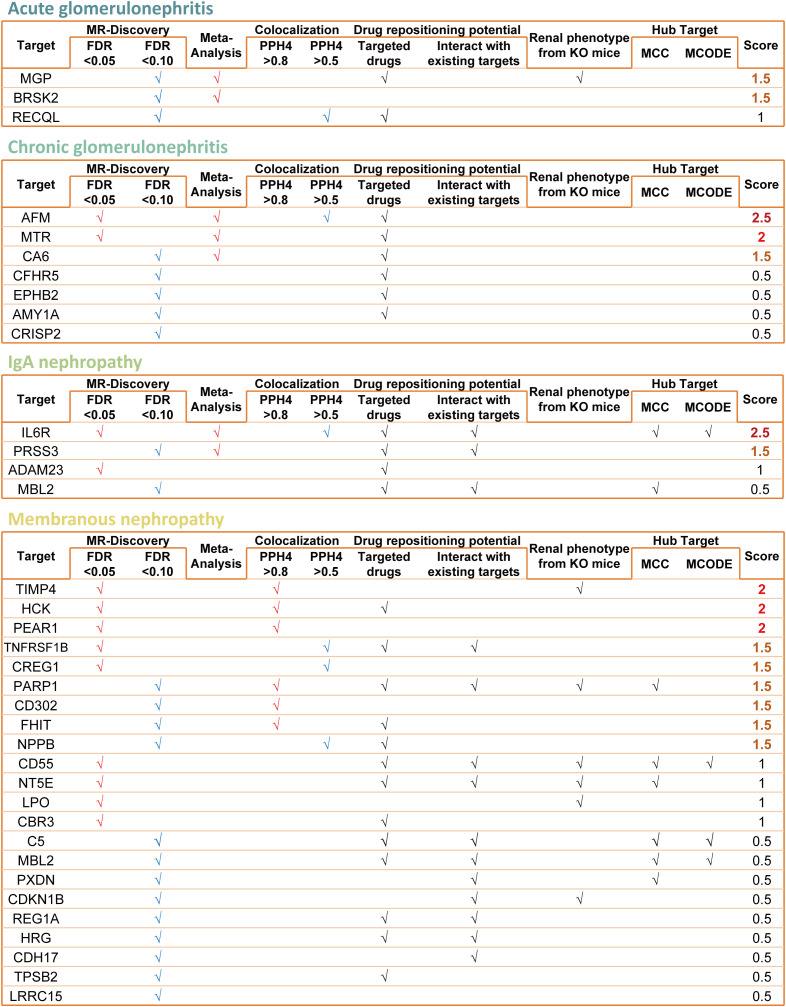
Ranking and summarizing the evidence levels for candidate targets.

To improve interpretability and address the large number of candidates across analyses, we summarized the strength of genetic evidence for each target using a simple rule-based prioritization scheme (Fig 7). Points were assigned based on the level of genetic support from each criterion. Specifically, targets received 1 point for each of the following: (i) an MR association meeting the high-confidence threshold (FDR < 0.05), (ii) strong Bayesian colocalization evidence consistent with a shared causal variant (PPH4 > 0.8), and (iii) supportive evidence from replication or meta-analysis in the FinnGen cohort. Targets received 0.5 points for more moderate genetic evidence, defined as an MR association at FDR < 0.10 and/or moderate colocalization support (0.5 < PPH4 ≤ 0.8). Importantly, downstream analyses—including network pharmacology, drug repurposing annotations, and renal phenotypes from knockout mouse models—were treated as descriptive and used to provide biological and translational context, but were not incorporated into the genetic evidence score.

Applying this evidence-based prioritization highlighted clear differences in the strength of genetic support across glomerulonephritis subtypes, with a maximum possible score of 3.0 reflecting convergence of independent genetic evidence components. In acute glomerulonephritis, no target accumulated high overall genetic support; *MGP* and *BRSK2* ranked highest with scores of 1.5, consistent with moderate genetic evidence in a relatively underpowered subtype. In chronic glomerulonephritis, *AFM* achieved the highest genetic evidence score (2.5), supported by a high-confidence MR association, replication/meta-analysis consistency, and moderate colocalization evidence. For IgA nephropathy, *IL6R* likewise reached a score of 2.5 based on high-confidence MR, replication/meta-analysis support, and moderate colocalization. In membranous nephropathy, several targets, including *HCK*, *TIMP4*, and *PEAR1*, accumulated scores of 2.0, reflecting convergent genetic support across multiple analyses, although no target achieved the maximum possible score (Fig 7).

## 4. Discussion

Our multi‑omics MR study integrated proteomic and transcriptomic quantitative trait loci with large‑scale genome‑wide association studies to prioritize druggable targets for four histologically defined glomerulonephritides. By applying stringent instrument selection, sensitivity analyses and Bayesian colocalization, we showed that several plasma proteins and transcripts have genetically supported associations on disease risk. Replication in independent FinnGen cohorts and interrogation of mouse knockout models and drug databases reinforced the translational relevance of the findings. We ranked the levels of evidence for the targets and summarized them in Fig 7. In the interpretation of our findings, greater weight was placed on high-confidence associations meeting the FDR < 0.05 threshold (as well as strong colocalization evidence), while results identified at FDR < 0.10 and moderate colocalization results were considered exploratory and intended to highlight potentially relevant pathways for further investigation. Compared with prior multi-omics or MR-based studies in glomerulonephritis, the present work extends existing approaches by systematically integrating proteomic and transcriptomic instruments across multiple GN subtypes, applying stringent colocalization and replication analyses, and explicitly linking genetic evidence to druggability, mouse phenotypes, and network-based prioritization.

### 4.1 Chronic glomerulonephritis

#### 4.1.1 Protective role of methionine synthase (MTR).

In the proteome‑wide MR, increased plasma levels of methionine synthase (*MTR*) were associated with lower risk of chronic glomerulonephritis (odds ratio [OR]≈0.56). MTR encodes a cobalamin‑dependent enzyme that regenerates methionine from homocysteine and plays a central role in one‑carbon metabolism. The *MTR* A2756G polymorphism has been studied in chronic kidney disease (CKD); individuals carrying the G allele showed a marginally decreased risk of CKD and a dose‑dependent protective effect [26]. Our MR results extend these observations to chronic glomerulonephritis and suggest that enhancing *MTR* activity may mitigate inflammatory kidney injury. Mechanistically, improved methionine synthesis could reduce homocysteine accumulation and oxidative stress while sustaining methylation reactions that support renal homeostasis.

#### 4.1.2 Afamin and other risk transcripts.

At the transcriptomic level, expression of *AFM* (encoding afamin) was associated with increased risk of chronic glomerulonephritis and showed moderate colocalization. Afamin is a vitamin E–binding glycoprotein in the albumin family; urine afamin concentrations are markedly elevated in patients with membranous nephropathy and IgA nephropathy [27]. High urine afamin and afamin/creatinine ratio correlate with albuminuria, suggesting that afamin reflects tubular or glomerular damage [28,29]. Our MR findings indicate that increased *AFM* transcription may contribute to chronic glomerulonephritis rather than merely being a consequence of injury. Elevated transcripts of *CFHR5* (complement factor H‑related protein 5) and the receptor tyrosine kinase *EPHB2* were also associated with chronic glomerulonephritis and showed moderate correlation evidence. High circulating *FHR‑5* levels correlate with lower glomerular filtration rate and progression to end‑stage kidney disease in IgA nephropathy [30]; thus over‑expression of *CFHR5* may similarly drive complement‑mediated injury in chronic glomerulonephritis. *EPHB2* is up‑regulated and phosphorylated in fibrotic kidneys; mice lacking *Ephb2* exhibit less inflammation and fibrosis after ischemia–reperfusion injury [31], supporting its pathological role. For the other transcripts (e.g., *CA6*, *CRISP2*, *AMY1A*), moderate associations were observed, but little is known about their role in renal disease; these genes warrant experimental studies to explore mechanisms.

### 4.2 Membranous nephropathy

#### 4.2.1 Pro‑inflammatory kinases and protective lectins.

Membranous nephropathy displayed the largest number of significant targets. The strongest proteomic association was the hematopoietic cell kinase *HCK* (OR=2.26), which showed perfect colocalization. *HCK* encodes a Src family tyrosine kinase that mediates macrophage activation; diseased human kidneys and murine models show high *HCK* expression in pro‑inflammatory macrophages, and genetic deletion or pharmacologic inhibition reduces M1‑like polarization, renal inflammation and fibrosis [32]. Our MR results therefore converge with experimental data and highlight *HCK* as a potential therapeutic target to limit immune‑mediated podocyte injury in membranous nephropathy.

Two proteins showed protective associations: *CD302* (OR=0.56) and *CDKN1B* (OR=0.33). *CD302* is a C‑type lectin receptor expressed on myeloid cells that mediates endocytosis and phagocytosis [33]. Its protective effect in MR may reflect enhanced clearance of immune complexes or apoptotic debris in glomeruli, limiting inflammation. *CDKN1B* encodes p27kip1, a cyclin‑dependent kinase inhibitor that restrains cell proliferation. In experimental mesangial proliferative glomerulonephritis, p27 levels were high in resting glomeruli but decreased during mesangial cell proliferation; proliferation was accompanied by increased cyclin A/CDK2 activity and returned to baseline when p27 recovered [34]. *CDKN1B* is recognized as a biomarker of acute kidney injury and glomerulonephritis [34], and our MR results suggest that maintaining p27 activity may protect against podocyte injury and fibrosis.

#### 4.2.2 Transcriptomic candidates and complement regulators.

At the transcript level, many genes exhibited strong or moderate correlation evidence for membranous nephropathy. *TIMP4*, which encodes tissue inhibitor of metalloproteinases 4, showed a protective association and strong colocalization. A recent integrative omics study identified *TIMP4* as a top candidate; serum *TIMP4* was elevated in membranous nephropathy patients compared with controls and molecular docking suggested therapeutic potential [35]. Conversely, transcripts of *TNFRSF1B* (tumour necrosis factor receptor 2) were associated with higher disease risk. In mouse models, *TNFR2* deficiency or inhibition protected against glomerulonephritis; glomerular endothelial expression of *TNFR2* was essential for complement deposition and injury [36], supporting our findings.

Other transcriptomic hits included *LPO*, *PARP1*, *PEAR1*, *MBL2*, *C5*, *CD55*, *NT5E*, *PXDN*, and *FHIT*. *LPO* encodes lactoperoxidase, and global deletion of the gene in mice leads to leukocyte infiltration, thickening of the glomerular basement membrane and collagen deposition in the renal cortex [37]—pathological hallmarks of glomerulonephritis—supporting our finding that high *LPO* expression increases risk. *PARP1* (poly [ADP‑ribose] polymerase 1) is a nuclear enzyme regulating DNA repair, and can accelerate ferroptosis-induced vascular calcification by activating Adora2a/Rap1 signaling [38,39]; its causal role in membranous nephropathy is unexplored, but PARP inhibitors have renoprotective effects in preclinical models [40], suggesting therapeutic potential. *PEAR1* (platelet endothelial aggregation receptor 1) transcripts were protective; although primarily expressed in platelets and endothelial cells, its role in podocyte‑endothelial crosstalk warrants further study [41]. *MBL2* encodes mannose‑binding lectin. In IgA nephropathy, both MBL deficiency and high MBL levels are associated with poor outcomes; the promoter contains IL‑6–responsive elements and variants influence MBL expression [42]. Our MR and colocalization results suggest that moderate expression of *MBL2* may be protective whereas extremes in either direction predispose to complement activation. *C5* transcripts were protective; this is consistent with the clinical efficacy of the *C5* inhibitor ravulizumab in atypical haemolytic uremic syndrome and ongoing trials in membranous nephropathy. *CD55* encodes decay‑accelerating factor; CD55‑deficient mice develop more severe anti‑glomerular basement membrane glomerulonephritis with increased complement activation [43]. *NT5E* encodes ecto‑5′‑nucleotidase (CD73), which dephosphorylates extracellular AMP; deficiency leads to vascular calcification and might exacerbate glomerular injury, inflammation, and fibrosis [44,45]. *PXDN* encodes peroxidasin, a heme‑containing peroxidase important for collagen IV cross‑linking. Studies have shown that the absence of *PXDN* reduces renal fibrosis [46]; Its downregulation has a nephroprotective effect in MR. *FHIT* (fragile histidine triad) is a tumour suppressor gene; strong colocalization suggests a role in preserving podocyte integrity [47]. These findings point to the complement cascade, extracellular matrix remodeling and endothelial–immune interactions as key pathways in membranous nephropathy.

### 4.3 IgA nephropathy

#### 4.3.1 Interleukin‑6 signalling and complement lectin pathway.

In the transcriptome‑wide analysis of IgA nephropathy, *IL6R* was a significant risk factor and showed moderate colocalization. IgA nephropathy is characterized by mesangial deposition of galactose‑deficient IgA1 and subsequent activation of inflammatory pathways [48]. Polymeric IgA1 stimulates mesangial cells to proliferate and secrete interleukin‑6 (IL‑6); complement activation further increases IL‑6 production, and blocking IL‑6 generation in a mesangioproliferative glomerulonephritis model protected against renal injury and reduced Th17 cytokine production [49]. A common *IL6R* variant (rs4845625) has been associated with susceptibility to chronic kidney disease; carriers of the C allele had higher serum creatinine and lower estimated glomerular filtration rate, with an odds ratio ~ 1.49 [50]. Our Mendelian randomization results provide genetically supported associations for the role of IL‑6/IL6R signalling in IgA nephropathy.

Additional transcriptomic associations included *ADAM23* (protective) and *PRSS3* and *MBL2* (risk). *ADAM23* encodes a disintegrin and metalloprotease domain‑containing protein involved in cell adhesion [51]; its protective association suggests a role in maintaining glomerular barrier integrity. *PRSS3* encodes trypsin‑3; increased expression could contribute to proteolytic damage of the mesangial matrix [52]. *MBL2* transcripts were associated with risk in IgA nephropathy, consistent with data showing that both low and high MBL levels contribute to disease progression [42]. Altogether, these genes highlight the importance of cytokine signalling and the complement lectin pathway in IgA nephropathy pathogenesis.

### 4.4 Acute glomerulonephritis

The transcriptome‑wide analysis identified three candidate genes for acute glomerulonephritis: *RECQL* and *BRSK2* (risk) and *MGP* (protective). *MGP* encodes matrix Gla protein, a vitamin K–dependent inhibitor of vascular calcification. Kidney injury up‑regulates *MGP* expression in endothelial and tubular cells, and impaired *MGP* expression aggravates capillary rarefaction and myofibroblast accumulation [53]. Thus, higher *MGP* transcription may limit acute glomerular damage by protecting the microvasculature. Evidence linking *RECQL* (a DNA helicase) and *BRSK2* (a brain‑specific serine/threonine kinase) to kidney disease is scarce; their associations may reflect pleiotropic effects or novel pathways requiring functional validation. The absence of significant proteomic associations for acute glomerulonephritis suggests that transcriptomic regulation plays a more prominent role in acute injury.

### 4.5 Strengths and limitations

Our study leverages several strengths. First, we integrated the largest available pQTL and eQTL datasets with four independent GN GWASs, enabling comprehensive evaluation of both plasma proteins and gene expression. Restricting instruments to cis‑acting variants and excluding the major histocompatibility complex minimized pleiotropy. Sensitivity analyses (MR‑Egger, MR‑PRESSO, heterogeneity tests) and Bayesian colocalization were conducted to ensure robustness. Replication in FinnGen cohorts, knockout phenotypes and drug databases provided orthogonal validation. Finally, network pharmacology illustrated how candidate targets interact with existing therapies and highlighted enriched pathways, underscoring translational potential.

An important observation is that the majority of prioritized targets with convergent evidence from MR, colocalization, mouse knockout phenotypes, and drug repurposing analyses were identified in membranous nephropathy. This may reflect the relatively well-defined immune- and complement-mediated pathogenesis of membranous nephropathy, which renders circulating proteins and immune regulators particularly amenable to drug-target MR approaches. In contrast, acute and chronic glomerulonephritides are more heterogeneous and may involve context-dependent or injury-driven transcriptional responses that are less readily captured by baseline pQTL instruments.

Several limitations warrant consideration. First, although Mendelian randomization reduces confounding, it relies on assumptions of linearity and that genetic instruments influence the outcome exclusively through the exposure. Residual horizontal pleiotropy cannot be completely excluded, particularly for genes with a limited number of instrumental variables. Associations based on single or very few cis instruments are inherently less robust and should be interpreted as exploratory unless supported by convergent evidence such as colocalization or independent replication. Second, all analyses were conducted in populations of European ancestry, and genetic architectures, allele frequencies, and effect sizes may differ across ancestries, which may limit the transferability of effect estimates to non-European populations. Future studies should be conducted to evaluate the universality of the multi-ancestry model. Third, statistical power varied substantially across glomerulonephritis subtypes. The relatively small sample sizes for acute and chronic glomerulonephritis constrained the ability to detect variants with modest effect sizes, which likely contributed to the absence of FDR-significant associations for acute glomerulonephritis. Specifically, smaller case numbers reduce statistical power by increasing the uncertainty of effect estimates and limiting the ability to overcome multiple-testing correction, thereby increasing the likelihood of false-negative findings. Therefore, null findings in these subtypes should be interpreted with caution and do not exclude the presence of biologically relevant but smaller-effect causal mechanisms. Fourth, although this study integrates multiple layers of genetic and in-silico evidence, including Mendelian randomization, colocalization, and drug annotation, the identified targets have not been functionally validated in experimental systems. Therefore, the designation of these targets as potential should be interpreted as a prioritization for downstream investigation rather than definitive therapeutic validation. Experimental studies, including CRISPR-based gene perturbation, in vitro cellular assays, and in vivo disease models, will be essential to confirm causal mechanisms, delineate cell-type–specific effects, and evaluate therapeutic feasibility.

In addition, transcriptomic instruments were derived from whole-blood eQTL data, which may not fully capture gene regulatory effects specific to renal cell types such as podocytes, mesangial cells, or tubular epithelial cells. Consequently, localized pathogenic mechanisms confined to the kidney may be underrepresented. However, glomerulonephritis is fundamentally an immune-mediated disease, in which systemic immune activation, circulating cytokines, and complement components play central pathogenic roles. Many of the prioritized targets, including IL6R, MBL2, and complement-related genes, are predominantly regulated in immune cells and act through circulating mediators rather than kidney-restricted expression. Moreover, kidney-specific eQTL resources remain limited in sample size and cell-type resolution, constraining their utility for large-scale MR analyses. By restricting instruments to cis-eQTLs and applying Bayesian colocalization, we aimed to mitigate tissue-mismatch bias and strengthen causal inference. Nonetheless, future studies integrating kidney tissue–specific and single-cell eQTL datasets will be required to refine cell-type–specific mechanisms and complement the systemic signals identified here. Finally, while colocalization supports the presence of a shared causal variant, it does not guarantee that the protein or transcript itself is the functional effector. Therefore, the identification of potential targets in this study should be interpreted as a prioritization step rather than definitive therapeutic validation.

## 5. Conclusions

In conclusion, our multi‑omics MR study identifies several candidate proteins and transcripts that causally influence glomerulonephritis susceptibility. The results implicate pathways such as one‑carbon metabolism (*MTR*), macrophage activation (*HCK*), cyclin‑dependent kinase inhibition (*CDKN1B*), complement regulation (*CFHR5*, *MBL2*, *C5*, *CD55*), metalloproteinase inhibition (*TIMP4*), cytokine signalling (*IL6R*), and vascular calcification (*MGP*). These findings offer mechanistic insights and nominate targets for therapeutic intervention or biomarker development. Prioritized genes with strong colocalization and supporting literature, such as *HCK*, *CD302*, *CDKN1B*, *TIMP4*, *TNFRSF1B*, *LPO*, *CD55* and *IL6R,* warrant further validation in cellular and animal models and, where drugs exist (e.g., HCK inhibitors or IL‑6 receptor blockers), clinical trials. Moreover, genes with limited evidence (e.g., *RECQL*, *BRSK2*, *PEAR1*) open avenues for discovery of novel pathways in glomerular biology. Future studies should incorporate diverse ancestries, examine longitudinal expression changes, and integrate single‑cell omics to elucidate cell‑type‑specific effects.

## Supporting information

S1 Table Details of dataset. (XLSX)

S2 Table SNPs excluded by MR-PRESSO. (XLSX)

S3 Table Results for proteomic-wide MR. (XLSX)

S4 Table Results for transcriptomic-wide MR. (XLSX)

S5 Table Results for heterogeneity test (pQTL). (XLSX)

S6 Table Results for pleiotropy test (pQTL). (XLSX)

S7 Table Results for heterogeneity test (eQTL). (XLSX)

S8 Table Results for pleiotropy test (eQTL). (XLSX)

S9 Table Existing drugs and targets for IGA glomerulonephritis. (XLSX)

S10 Table Existing drugs and targets for membranous glomerulonephritis. (XLSX)
